# Design, Synthesis, and In Vitro and In Vivo Biological Studies of a 3′-Deoxythymidine Conjugate that Potentially Kills Cancer Cells Selectively

**DOI:** 10.1371/journal.pone.0052199

**Published:** 2012-12-26

**Authors:** Qiong Wei, Dejun Zhang, Anna Yao, Liyi Mai, Zhiwei Zhang, Qibing Zhou

**Affiliations:** 1 Institute of Materia Medica, College of Life Science and Technology, Huazhong University of Science and Technology, Wuhan, Hubei, China; 2 Hepatic Surgery Center, Tongji Hospital, Tongji Medical College, Huazhong University of Science and Technology, Wuhan, Hubei, China; 3 Department of Medicinal Chemistry, Virginia Commonwealth University, Richmond, Virginia, United States of America; Univ of Bradford, United Kingdom

## Abstract

Thymidine kinases (TKs) have been considered one of the potential targets for anticancer therapeutic because of their elevated expressions in cancer cells. However, nucleobase analogs targeting TKs have shown poor selective cytotoxicity in cancer cells despite effective antiviral activity. 3′-Deoxythymidine phenylquinoxaline conjugate (dT-QX) was designed as a novel nucleobase analog to target TKs in cancer cells and block cell replication via conjugated DNA intercalating quinoxaline moiety. In vitro cell screening showed that dT-QX selectively kills a variety of cancer cells including liver carcinoma, breast adenocarcinoma and brain glioma cells; whereas it had a low cytotoxicity in normal cells such as normal human liver cells. The anticancer activity of dT-QX was attributed to its selective inhibition of DNA synthesis resulting in extensive mitochondrial superoxide stress in cancer cells. We demonstrate that covalent linkage with 3′-deoxythymidine uniquely directed cytotoxic phenylquinoxaline moiety more toward cancer cells than normal cells. Preliminary mouse study with subcutaneous liver tumor model showed that dT-QX effectively inhibited the growth of tumors. dT-QX is the first molecule of its kind with highly amendable constituents that exhibits this selective cytotoxicity in cancer cells.

## Introduction

With cancers being the leading cause of death world-wide, developing safe and effective anticancer agents remains in urgent need. Molecularly targeted therapy has been the recent focus for anticancer drug development, as seen in the example of Sorafenib [1], [2]. Sorafenib is a multi-tyrosine kinase inhibitor that can potentially minimize adverse effects such as hepatotoxicity caused by other anticancer drugs including 5-fluorouracil and doxorubicin [3], [4]. Similar to tyrosine kinases, thymidine kinases (TKs) have been considered another potential anticancer target [5]–[8]. TKs are the first phosphorylating enzymes in the thymidine salvage pathway converting thymidine to its 5′-phosphate form for DNA synthesis. In normal mammalian cells, cytosolic TKs are only present at a low level in the S phase of cells; whereas elevated level of TKs have been observed in virus infected cells and rapidly proliferating cancer cells [5]–[8], e.g., lung tumors and breast cancer tissues [9], [10].

Nucleobase analogs targeting TKs such as AZT and acyclovir have been shown effective antiviral activity [11], [12]. However, poor cancer-selectivity and strong side effects have been associated with nucleobase analogs including neutron capture agent 5-thymidine boron conjugate and radioisotopic iodinated deoxyuridine [13], [14]. Recently, a combination therapy using predelivered thymidine kinase followed by nucleobase analogs has been investigated in liver cancer patients with limited effects achieved [15]. This is likely due to that TKs oriented nucleobase analogs such as AZT are quickly removed by nucleobase repair processes after they are incorporated in the DNA synthesis of cancer cells via the thymidine salvage pathway [16]–[18]. 5-Fluorouracil, another thymine analog, is a dihyrofolate reductase inhibitor and directly causes cytotoxicity in normal hepatocytes [3], [19]. Therefore, alternative design of more effective nucleobase analogs targeting TKs is needed.

**Figure 1:**
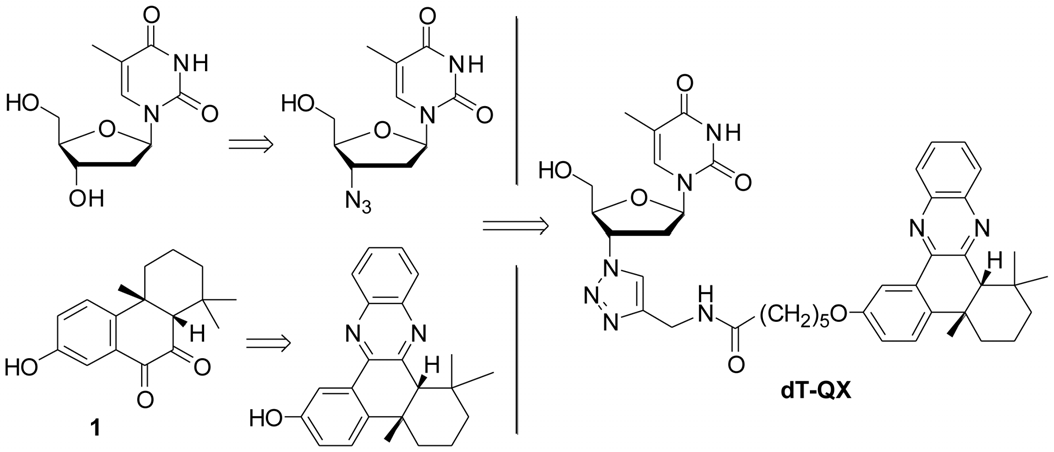
Structural design of dT-QX.

We report here a novel 3′-deoxythymidine phenylquinoxaline conjugate (dT-QX, Figure 1) that selectively kills a variety of cancer cells, but not normal hepatocytes. Structurally, dT-QX is a 3′-triazole-3′-deoxythymidine linked to a DNA intercalating quinoxaline moiety. dT-QX was designed to target the thymidine salvage pathway based on the fact that 3′-triazole thymidine derivatives are recognized by human thymine kinases [20], [21]. The purpose of adding the quinoxaline moiety into dT-QX structure was to block cellular removal of thymidine analogs [16]–[18] via DNA intercalation in the DNA synthesis of cancer cells, because quinoxaline moiety is a known DNA intercalator [22]. In addition, the quinoxaline structure can be conveniently synthesized by one step condensation of a diketone compound **1** [23] and an ortho-phenylenediamine (Figure 1). More importantly, the quinoxaline structure is highly amendable for chemical modifications with a variety of substituents for advanced structure activity study to optimize the potential biological activity. The anticancer activity of dT-QX was then evaluated using a panel of cancer cells. The effects of dT-QX on inhibiting DNA synthesis and inducing cellular oxidative stress were also investigated. Finally, the inhibition of tumor growth by dT-QX was assessed in mice bearing subcutaneous liver tumors.

## Materials and Methods

### Synthesis

All chemicals were purchased from Sigma-Aldrich (WI, USA), J&K Scientific Ltd. (Beijing, China), or Sinopharm Chemical Reagent Co., Ltd (Shanghai, China) and used without further purification. NMR spectra were recorded with Bruker Avance-400 NMR spectrometer (Madison, WI, USA). Abbreviations used for the split patterns of proton NMR signals are: singlet (s), doublet (d), triplet (t), quartet (q), quintet (qui), multiplet (m) and broad signal (br). Electrospray ionization mass spectroscopy (ESI-MS) analysis was carried out with a Thermo Fisher TSQ Quantum Max Triple Stage Quadrupole mass spectrometer (MA, USA). Compound **1** was obtained as previously reported [23].

#### 6-Bromo-*N*-(prop-2-ynyl)hexanamide (2)

To a solution of 6-bromohexanoic acid (694 mg, 3.56 mmol) in dry CH_2_Cl_2_ (50 mL) were added propargylamine hydrochloride (300 mg, 3.28 mmol), triethylamine (332 mg, 3.28 mmol) and 1-(3-dimethylaminopropyl)-3-ethylcarbodiimide hydrochloride (630 mg, 3.28mmol). The reaction mixture was stirred under N_2_ for 16 h at room temperature. The resulting solution was extracted with CH_2_Cl_2_ (150 mL×3). The organic layers were collected, washed with brine (250 mL), dried with MgSO_4_ and concentrated. A flash chromatographic separation (0–40% EtOAc in hexane) afforded **2** as 1∶1 syn/anti-amide mixture as a colorless oil (390 mg, 1.68 mmol) in 51% yield. ^1^H NMR (CDCl_3_, 400 MHz, 1∶1 syn/anti): *δ* = 5.71 (br s, 1H; NH), 4.05 (dd, *^3^J*(H,H) = 5.2 Hz, *^4^J*(H,H) = 2.5 Hz, 2H; CH_2_), 3.53 (t, ^3^ *J*(H,H) = 6.6 Hz, 1H; 0.5CH_2_), 3.40 (t, ^3^ *J*(H,H) = 6.7 Hz, 1H; 0.5CH_2_), 2.24-2.19 (m, 3H; CH, CH_2_), 1.87(qui, *^3^J*(H,H) = 7.2 Hz, 1H; 0.5CH_2_), 1.79 (qui, *^3^J*(H,H) = 7.0 Hz, 1H; 0.5CH_2_), 1.67 (qui, *^3^J*(H,H) = 7.52 Hz, 2H; CH_2_), 1.50-1.41 ppm (m, 2H; CH_2_); ^13^C NMR (CDCl_3_, 100.6 MHz, 1∶1 syn/anti): *δ* = 172.3, 172.2, 79.5, 71.6, 44.8, 36.1, 36.1, 33.5, 32.4, 32.2, 29.1, 27.7, 26.4, 24.7, 24.5 ppm (Figure S1); HRMS calcd for C_9_H_15_BrNO [M+H]^+^232.0337 and [M+2+H]^+^234.0316, found 232.0324 and 234.0413.

#### *N*-(Prop-2-ynyl)-6-(4b,8,8-trimethyl-9,10-dioxo-4b,5,6,7,8,8a,9,10-octahydrophenanthren-2-yloxy)hexanamide (3)

To a solution of **2** (390 mg, 1.68 mmol) in dry DMF (50 mL) were added **1** (355 mg, 1.30 mmol) and potassium carbonate (1.8 g, 13.0 mmol). The reaction mixture was stirred under N_2_ for 16 h at room temperature and then acidified to pH 5 with 5 N HCl. The resulting solution was extracted with CH_2_Cl_2_ (150 mL×3). The organic layers were collected, washed with brine (250 mL), dried with MgSO_4_ and concentrated. A flash chromatographic separation (0–40% EtOAc in hexane) afforded **3** as a yellow oil (320 mg, 0.76 mmol) in 58% yield. ^1^H NMR (CDCl_3_, 400 MHz): *δ* = 7.53 (d, *^4^J*(H,H) = 2.9 Hz, 1H; CH), 7.35 (d, *^3^J*(H,H) = 8.8 Hz, 1H; CH), 7.21 (dd, *^3^J*(H,H) = 8.7 Hz, *^4^J*(H,H) = 2.7 Hz, 1H; CH), 5.94 (br s, 1H; NH), 4.04 (dd, *^3^J*(H,H) = 5.3 Hz, *^4^J*(H,H) = 2.6 Hz, 2H; CH_2_), 4.00 (t, *^3^J*(H,H) = 6.4 Hz, 2H; CH_2_), 2.65 (s, 1H; CH), 2.53 (d, *^3^J*(H,H) = 14.3 Hz, 1H, CH), 2.24 (t, *^3^J*(H,H) = 7.6 Hz, 2H; CH_2_), 2.21 (d, *^4^J*(H,H) = 2.5 Hz, 1H; CH), 1.82-1.70 (m, 4H; 2CH_2_), 1.56-1.31(m, 7H; 3CH_2_, CH), 1.18 (s, 3H; CH_3_), 0.95 (s, 3H; CH_3_), 0.38 ppm (s, 3H; CH_3_); ^13^C NMR (CDCl_3_, 100.6 MHz): *δ* = 199.0, 181.3, 172.4, 158.1, 142.6, 134.6, 126.1, 124.2, 112.6, 71.7, 68.9, 68.1, 41.9, 39.2, 39.0, 36.3, 36.3, 35.5, 31.4, 29.7, 29.2, 28.9, 25.7, 25.2, 24.3, 18.8 ppm (Figure S2); HRMS calcd for C_26_H_34_NO_4_ [M+H]^+^424.2488, found 424.2487.

#### *N*-(Prop-2-ynyl)-6-(4b,8,8-trimethyl-4b,5,6,7,8,8a-hexahydrodibenzo[a,c]phenazin-2-yloxy)-hexanamide (4)

To a solution of **3** (320 mg, 0.76 mmol) in toluene (25 mL) were added *o*-phenylenediamine (103 mg, 0.95 mmol) and silica gel (300 mg). The reaction mixture was refluxed under N_2_ for 18 h and then concentrated. A flash chromatographic separation (0–40% EtOAc in hexane) afforded **5** as a yellow solid (230 mg, 0.46 mmol) in 61% yield. ^1^H NMR (CDCl_3_, 400 MHz): *δ* = 8.09-8.07 (m, 3H; 3CH), 7.73-7.67 (m, 2H; 2CH), 7.30 (d, *^3^J*(H,H) = 8.8 Hz, 1H; CH), 7.02 (dd, *^3^J*(H,H) = 8.4 Hz, *^4^J*(H,H) = 2.8 Hz, 1H; CH), 5.71 (br s, 1H; NH), 4.14-4.06 (m, 4H; 2CH_2_), 2.88 (s, 1H; CH), 2.56 (br d, *^3^J*(H,H) = 14 Hz, 1H, CH), 2.28-2.23 (m, 3H; CH_2_, CH), 1.89-1.73 (m, 4H; 2CH_2_), 1.59-1.47 (m, 7H; 3CH_2_, CH), 1.02 (s, 3H; CH_3_), 0.99 (s, 3H; CH_3_), 0.16 ppm (s, 3H; CH_3_); ^13^C NMR (CDCl_3_, 100.6 MHz): *δ* = 172.4, 158.0, 154.8, 148.4, 141.9, 141.3, 137.6, 134.8, 129.2, 129.1, 129.0, 128.6, 125.0, 118.1, 111.2, 79.6, 71.6, 67.7, 59.7, 42.0, 37.3, 36.3, 36.1, 36.0, 34.8, 31.5, 29.2, 29.1, 25.8, 25.3, 22.1, 19.0 ppm (Figure S3); HRMS calcd for C_32_H_38_N_3_O_2_ [M+H]^+^496.2964, found 496.2964.

#### *N*-((1-(2-(Hydroxymethyl)-5-(5-methyl-2,4-dioxo-3,4-dihydropyrimidin-1(2H)-yl)-tetrahydrofuran-3-yl)-1H-1,2,3-triazol-4-yl)methyl)-6-(4b,8,8-trimethyl-4b,5,6,7,8,8a-hexahydrodibenzo[a,c]phenazin-2-yloxy)hexanamide (dT-QX)

To a solution of **4** (100 mg, 0.2 mmol) in 5 mL DMF and 10 mL CH_2_Cl_2_ were added 3′-azido-3′-deoxythymidine (54 mg, 0.20 mmol) and an aqueous solution of sodium ascorbate (40 mM, 15 mL). The reaction mixture was purged with N_2_ for 10 min, and CuSO_4_·5H_2_O (8 mg, 0.03 mmol) was added. The reaction mixture was then stirred under N_2_ at room temperature for 12 h. The resulting solution was extracted with CH_2_Cl_2_ (150 mL×3). The organic layers were collected, washed with brine (200 mL), dried with MgSO_4_ and concentrated. A flash chromatographic separation (0–7% MeOH in CH_2_Cl_2_) afforded dT-QX as a white solid (114 mg, 0.15 mmol) in 75% yield. ^1^H NMR (DMSO-*d_6_*, 400 MHz): *δ* = 11.35 (s, 1H; OH), 8.34 (t, ^3^ *J*(H,H) = 5.5 Hz, 1H; CH), 8.14-8.11 (m, 2H; 2CH), 8.06-8.04 (m, 1H; CH), 7.96 (d, ^4^ *J*(H,H) = 2.8 Hz, 1H; CH), 7.79 (m, 3H; 2CH, NH), 7.39 (d, ^3^ *J*(H,H) = 8.7 Hz, 1H; CH), 7.11 (dd, ^3^ *J*(H,H) = 8.5 Hz, ^4^ *J*(H,H) = 2.8 Hz, 1H; CH), 6.40 (t, ^3^ *J*(H,H) = 6.6 Hz, 1H; CH), 5.36-5.33 (m, 1H; CH), 5.28 (t, ^3^ *J*(H,H) = 5.2 Hz, 1H; NH), 4.31 (d, ^3^ *J*(H,H) = 5.5 Hz, 2H; CH_2_), 4.18 (m, 1H; CH), 4.06 (t, ^3^ *J*(H,H) = 6.4 Hz, 2H; CH_2_), 3.67-3.60 (m, 2H; CH_2_), 2.85 (s, 1H; CH), 2.67-2.57 (m, 2H; CH_2_), 2.16 (t, ^3^ *J*(H,H) = 7.4 Hz, 2H; CH_2_), 1.79-1.62 (m, 5H; CH_3_, CH_2_), 1.56-1.43 (m, 10H; 5CH_2_), 0.93 (s, 3H; CH_3_), 0.91 (s, 3H; CH_3_), 0.07 ppm (s, 3H; CH_3_); ^13^C NMR (DMSO-*d_6_*, 100.6 MHz): *δ* = 172.0, 163.6, 157.4, 154.2, 150.4, 147.5, 145.2, 141.1, 140.9, 137.1, 136.1, 134.1, 129.5, 129.4, 128.9, 128.4, 125.3, 122.4, 117.7, 110.8, 109.5, 84.4, 83.8, 67.4, 60.6, 59.0, 58.5, 41.2, 37.0, 36.8, 35.5, 35.1, 34.9, 34.4, 34.0, 31.3, 28.5, 25.2, 24.9, 21.6, 18.5, 12.2 ppm; HRMS calcd for C_42_H_51_N_8_O_6_ [M+H]^+^763.3932, found 763.3959 (Figures S4 and S5).

#### 4b,8,8-Trimethyl-9,10-dioxo-4b,5,6,7,8,8a,9,10-octahydrophenanthren-2-yl 4-methyl-piperazine-1-carboxylate (5)

To a solution of **1** (650 mg, 2.39 mmol) in DMF (30 mL) were added 4-methyl-1-piperazinecarbonyl chloride hydrochloride (630 mg, 3.16 mmol) and potassium carbonate (3.4 g, 24 mmol). The reaction mixture was stirred under N_2_ for 18 h and then acidified to pH 5 with 5 N HCl. The resulting solution was extracted with CH_2_Cl_2_ (150 mL×3). The organic layers were collected, washed with brine (250 mL), dried with MgSO_4_ and concentrated. A flash chromatographic separation (0–25% EtOAc in hexane) afforded **5** as a yellow oil (730 mg, 1.83 mmol) in 77% yield. ^1^H NMR (CDCl_3_, 400 MHz): *δ = *7.86 (s, 1H; CH), 7.49 (s, 2H; 2CH), 3.71 (br s, 2H; CH_2_), 3.61 (br s, 2H; CH_2_), 2.69 (s, 1H; CH), 2.58 (d, ^3^ *J*(H,H) = 13.3 Hz, 1H; CH), 2.49 (br s, 4H; 2CH_2_), 2.37 (s, 3H; CH_3_), 1.47-1.42 (m, 5H; 2CH_2_, CH), 1.23 (s, 3H; CH_3_), 0.98 (s, 3H; CH_3_), 0.41 ppm (s, 3H; CH_3_); ^13^C NMR (CDCl_3_, 100.6 MHz): δ 198.2, 180.6, 152.5, 149.8, 147.4, 134.6, 129.4, 126.3, 122.5, 68.7, 53.7, 53.6, 41.7, 39.5, 38.7, 36.2, 36.1, 35.5, 31.2, 26.9, 24.2, 24.2, 18.7 ppm (Figure S6); HRMS calcd for C_23_H_31_N_2_O_4_ [M+H]^+^299.2284, found 299.2294.

#### 4b,8,8-Trimethyl-4b,5,6,7,8,8a-hexahydrodibenzo[a,c]phenazin-2-yl 4-methylpiperazine-1-carboxylate (Pip-QX)

To a solution of **5** (330 mg, 0.83 mmol) in toluene (25 mL) were added *o*-phenylenediamine (100 mg, 0.92 mmol) and silica gel (300 mg). The reaction mixture was refluxed under N_2_ for 18 h and then concentrated. A flash chromatographic separation (0–3% MeOH in CH_2_Cl_2_) afforded Pip-QX as a yellow oil (310 mg, 0.66 mmol) in 79% yield. ^1^H NMR (DMSO-*d_6_*, 400 MHz): *δ* = 8.14-8.05 (m, 3H; 3CH), 7.81-7.79 (m, 2H; 2CH), 7.49 (d, ^3^ *J*(H,H) = 8.4 Hz, 1H; CH), 7.29 (dd, ^3^ *J*(H,H) = 8.4 Hz, ^4^ *J*(H,H) = 2.4 Hz, 1H; CH), 3.63 (br s, 2H; CH_2_), 3.46 (br s, 2H; CH_2_), 2.88 (s, 1H; CH), 2.58 (m, 1H; CH_2_), 2.38 (br s, 4H; 2CH_2_), 2.16 (s, 3H; CH_3_), 1.51-1.38 (m, 5H; 2CH_2_, CH), 0.96 (s, 3H; CH_3_), 0.90 (s, 3H; CH_3_), 0.06 ppm (s, 3H; CH_3_); ^13^C NMR (DMSO-*d_6_*, 100.6 MHz): *δ* = 154.4, 153.3, 150.5, 147.5, 142.3, 141.6, 134.7, 130.2, 130.2,129.4, 129.0, 125.7, 125.2, 119.3, 58.9, 54.7, 54.6, 46.2, 44.6, 44.0, 41.6, 37.7, 36.1, 35.4, 34.7, 31.8, 22.2, 19.0 ppm (Figure S7); HRMS calcd for C_29_H_35_N_4_O_2_ [M+H]^+^471.2760, found 471.2767.

### Biological Studies

The animal protocol was approved by the Reviewing Committee of College of Life Science and Technology at Huazhong University of Science and Technology. Cancer cell lines HepG2, Hep3B, MCF-7 and C6 were obtained from ATCC (VA, USA). Human liver cells HL-7702 and murine liver cancer cells H22 were from Shanghai Institute of Life Science Cell Culture Center (Shanghai, China). Cells were maintained in high glucose DMEM or RPMI-1640 medium (Invitrogen, CA, USA) supplemented with 10% heat-inactivated fetal bovine serum (FBS), 25 mM HEPES, 2 mM L-glutamine, 0.1 mM nonessential amino acids, 1.0 mM sodium pyruvate, 50 U/mL penicillin, and 50 µg/mL streptomycin at 37°C and 5% CO_2_. Stock solutions of dT-QX, Pip-QX or AZT were prepared in DMSO, and doxorubicin hydrochloride salt was directly dissolved in water. All biological chemicals were obtained from Sigma Aldrich (WI, USA) unless specified otherwise. All of the experiments were independently repeated at least three times.

#### Cell viability MTT assay

Cells (5,000 per well) were seeded on 96-well plates in growth media overnight before each treatment in triplicates. dT-QX, Pip-QX or AZT was at a final concentration of 0, 0.1, 1, 10, 20, 50, 100, or 200 µM with total DMSO less than 0.2%. Doxorubicin was used as a comparison at a final concentration of 0.05, 0.1, 0.2, 0.5, 1.0 or 2.0 µM. After 72 h, MTT assay was carried out as reported [23]. The cell viability of each treatment was plotted with GraphPad Prism program, and IC_50_ values were obtained using sigmoidal dose-response analysis provided by the software (version 4.00, GraphPad Software, CA, USA).

#### Anti-BrdU fluorescence assay

Hep3B, HepG2 or HL-7702 cells (50,000 per well) were seeded in the growth media overnight on 48-well plates and treated with 0, 10 or 50 µM dT-QX for 5 h. Anti-BrdU assay was carried out according to the manufacturer’s recommendation (BD Biosciences, NJ, USA). Briefly, BrdU solution (0.1 mg/mL) was added to each well, and cells were incubated at 37°C for 3 h. Cell medium was removed and cells were fixed with 3.7% formaldehyde in PBS. Cells were permeablized with 0.1% triton-100 in PBS and blocked with 3% FBS in PBS solution. Cellular DNA was denatured by DNaseI (0.3 mg/mL) in PBS solution. The incorporated BrdU was stained with Alexa Fluor®488 anti-BrdU monoclonal antibody (BD Biosciences, NJ, USA). The nuclei were counter-stained with Hoechst 33342 solution. Cell images of each treatment were captured with Olympus IX71 inverted microscope equipped with a digital camera under appropriate lights. The impacts of Pip-QX and AZT on the DNA synthesis in Hep3B were assessed similarly as described above.

#### Fluorescence study of mitochondrial superoxide production

Hep3B or HL-7702 cells (50,000 per well) were seeded in the growth media overnight on 48-well plates. Cells were treated with 0 or 50 µM dT-QX for 8 h and then medium was removed. A solution of MitoSOX Red mitochondrial superoxide indicator (Invitrogen, CA, USA) in PBS was added and incubated at 37°C for 20 min. Cells were then washed once with PBS and counter-stained with Hoechst 33342 solution. Fluorescent images were captured with Olympus IX71 inverted microscope under appropriate lights. The study with 100 µM Mn(III) tetrakis(1-methyl-4-pyridyl)porphyrin tetratosylate hydroxide (MnTmPyP, EMD Chemicals, Inc., USA) as a positive control on Hep3B cells was carried similarly.

#### Subcutaneous liver tumor study in mice

Murine liver cancer cells H22 were initially maintained in the RPMI-1640 growth medium and then grow in the BALB/c mice (Hubei Provincial Laboratory Animal Center, China) intraperitoneally. Mice were euthanized after 4 days and H22 cells were harvested with PBS solution. H22 cells were washed once with sterile PBS and were injected subcutaneously (3x10^6^ cells per mouse) at the lower back of naive BALB/c mice. Once tumors reached an average size of (8x8 mm, 340 mm^3^), nine mice were randomly divided into three groups (3 per group) and injected with 100 µL of saline (with 0.1% DMSO), AZT (50 µM in sterile PBS with 0.1% DMSO) or dT-QX (50 µM in sterile PBS with 0.1% DMSO) on the tumor site on day 1 and day 7. The tumor growth and body weight were monitored daily. On day 12 followed the first injection, all mice were euthanized and images of tumors were recorded. Statistic analysis (one way ANOVA with Tukey’s multiple comparison test) of the treatments was performed using GraphPad Prism software.

## Results and Discussion

**Figure 2:**
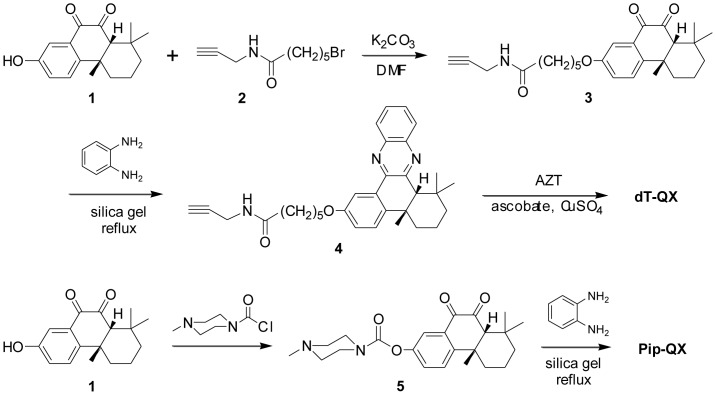
Synthesis of dT-QX and reference compound Pip-QX.

Synthesis of dT-QX was achieved by coupling AZT with phenylquinoxaline **4** via copper(I)-catalyzed click reaction (Figure 2). Intermediate **3** was obtained from nucleophilic substitution of bromoalkyne **2** with oxidized terpenone **1**. Phenylquinoxaline **4** was obtained by condensation with *o*-diaminobenzene and silica gel under reflux in toluene, which was much better than acetonitrile, DMF or ethanol. The conjugation of AZT with phenylquinoxaline **4** to dT-QX was accomplished in 75% yield using the in situ reduction of copper(II) by ascorbate [20], [21]. Phenylquinoxaline **4**, a reference molecule, was found to be poorly soluble in 1% DMSO aqueous solution and hence was modified to a *N*-methylpiperazine derivative Pip-QX as shown in Figure 2. Pip-QX was synthesized in a manner similar to dT-QX via conversion of **1** to the piperazine derivative **5**, followed by condensation with *o*-diaminobenzene in an overall yield of 61%. Pip-QX served as one of reference compounds in following biological studies. Multiple batches of dT-QX and control compounds were synthesized, and all the batches performed consistently not only in chemistry but also in biological studies.

**Figure 3:**
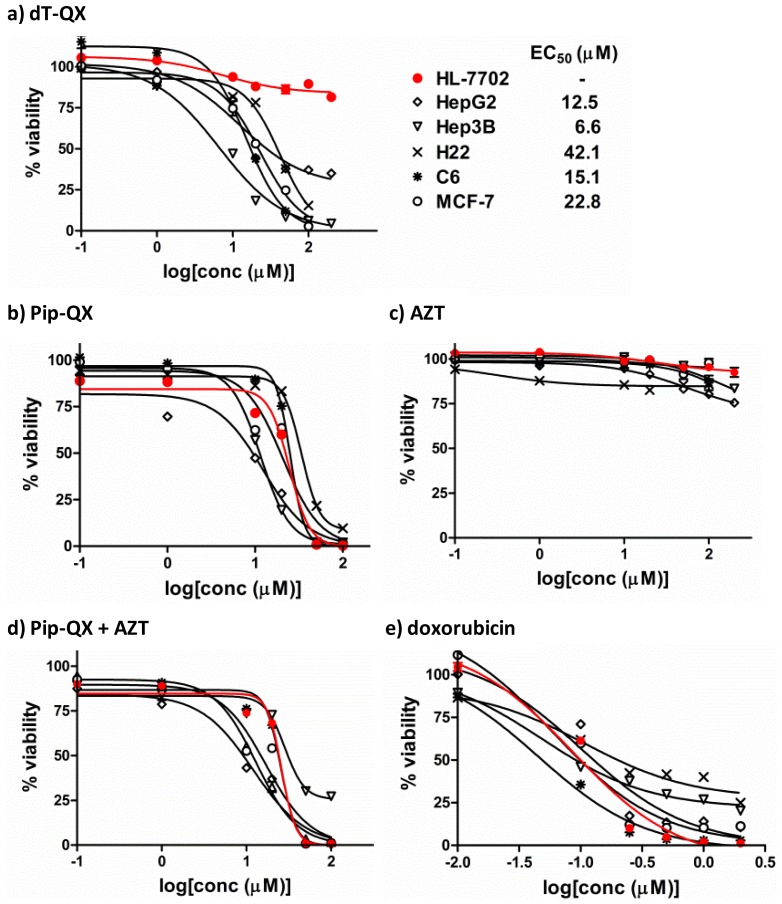
Selective killing of cancer cell lines over a normal human liver cell line. A panel of cancer cell lines (black color) and a normal liver hepatocyte cell line (red color) were treated with a) dT-QX; b) Pip-QX; c) AZT; d) Pip-QX plus AZT and e) doxorubicin, respectively. Cell viability was assessed after 72 h incubation by MTT assay. The percent viability was calculated and fitted with dose-response curves using GraphPad Prism software. All treatments were conducted in triplicates and repeated at least three times.

The biological activity of dT-QX was first assessed using cell viability assay on a panel of cancer cell lines including human liver carcinoma HepG2 and Hep3B, mouse liver carcinoma H22, breast adenocarcinoma MCF-7, and rat brain glioma C6 cells. Human liver HL-7702 cell line was used as a representative of normal cells in the study. HL-7702 cells are transformed human normal hepatocytes with low expression of cancer markers [24]. Treatment of HL-7702 with dT-QX did not resulted in any significant cytotoxicity at concentrations as high as 200 µM (Figure 3a). The EC_50_ of dT-QX on all cancer cells was found to range from 6.6 to 42.1 µM. The most pronounced cytotoxicity was observed on human hepatocellular carcinoma Hep3B cells with more than 80% cell death at 20 µM, followed by breast adenoma MCF-7 and brain glioma C6 cells. In stark contrast, Pip-QX non-selectively killed all cell lines including HL-7702 at 50 µM (Figure 3b). The second reference compound, AZT as a 3′-deoxythymidine analog, was found to exhibit low cytotoxicity against these cell lines (Figure 3c), while co-treatment of AZT plus Pip-QX also non-selectively killed all cell lines (Figure 3d), similar to that of Pip-QX treatment alone. These results suggested that the selective killing of cancer cells over normal liver cells by dT-QX was due to the unique covalent conjugation of cytotoxic phenylquinoxaline moiety with 3′-deoxythymidine. In comparison, the anticancer drug doxorubicin was found highly toxic to all these cells. In fact, doxorubicin was even more toxic toward normal liver 7702 cells than liver cancer Hep3B or H22 cells at concentrations above 0.5 µM (Figure 3e).

**Figure 4:**
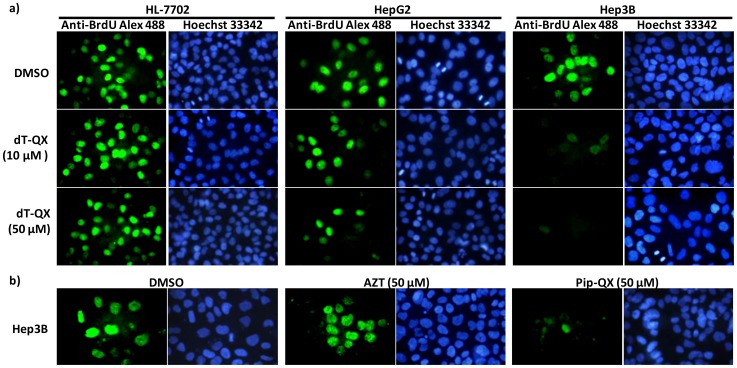
Fluorescence images of anti-BrdU assay of cell division and proliferation after compound treatment. BrdU assay was carried out after cells treated with compounds for 5 h. The incorporated BrdU was stained with anti-BrdU Alexa Fluor®488 monoclonal antibody (green color) with cell nuclei counter-stained with Hoechst 33342 (blue color). a) BrdU incorporation in HL-7702, HepG2 or Hep3B cells treated with DMSO or dT-QX; b) BrdU incorporation inHep3B cells treated with DMSO, AZT or Pip-QX.

To further understand the selective cytotoxicity of dT-QX towards cancer cells, we first investigated cell proliferation using anti-BrdU fluorescence assay. BrdU (5-bromo-3′-deoxyuridine) is a thymidine analog that is incorporated into cell genome as it replicates [25]. Thus, the level of BrdU in cell nucleus reflects the level of cell division and proliferation. Considering the above cell viability results, human HL-7702, HepG2 and Hep3B cells were investigated as representatives of normal and cancer cells. The anti-BrdU assay was carried out at 5 h after dT-QX treatment to allow enough accumulation of compound in cells and to determine its impact on cellular DNA synthesis. The level of BrdU in cell nuclei was measured using a fluorescent anti-BrdU conjugate [25] (green color) with cell nuclei counter-stained by Hoechst 33342 (blue color) as shown in Figure 4.

In normal human HL-7702 cells, treatments of DMSO and dT-QX resulted in similar levels of BrdU incorporation in cell nuclei, which indicated the presence of dT-QX did not interfere with cell division and proliferation. However, significant loss of green fluorescence was observed in cancerous HepG2 and Hep3B cells with dT-QX treatment as compared to that of DMSO control (Figure 4a). These results indicated that dT-QX selectively inhibited cellular DNA synthesis of HepG2 and Hep3B cells at the early stage of treatment resulting in selective killing both of cancer cells. The inhibition of BrdU incorporation was much more pronounced on Hep3B cells with the complete loss of green fluorescence in cell nuclei (Figure 4a). This was consistent with cell viability results that dT-QX was more effective at killing Hep3B than HepG2 cells. The more pronounced cytotoxicity on Hep3B cell than HepG2 may be due to the fact that Hep3B is originated from hepatitis B infection [26].

To elucidate which chemophore of dT-QX was responsible for the inhibition of DNA synthesis, the anti-BrdU fluorescence assay was carried out with AZT or Pip-QX on Hep3B cells (Figure 4b). Treatment of AZT resulted in no significant inhibition of DNA synthesis, while Pip-QX significantly inhibited the DNA synthesis in cells, similar to that of dT-QX, indicating phenylquaxinoline chemophore was responsible for the inhibition of DNA synthesis observed. In combination with cell viability results, it suggested that conjugation with 3′-deoxythymidine uniquely modified the cytotoxic phenylquinoxaline chemophore to make it more selective toward cancer cells than normal hepatocytes. These results also confirmed that conjugating the quinoxaline moiety with 3′-triazole-3′-deoxythymine led to selectively targeting cancer cells by blocking their DNA synthesis.

The selective inhibition of nuclear DNA synthesis by dT-QX in cancer cells led to the hypothesis that dT-QX might selectively inhibit the mitochondrial DNA synthesis and cause mitochondrial dysfunction as well. Unfortunately, the anti-BrdU fluorescent assay was not sensitive enough to observe such inhibition in cells. Alternatively, mitochondrial dysfunction due to DNA depletion by nucleoside analogs has been reported to occur concurrently with elevated superoxide level in mitochondria after treatment for 4 days [27]. Therefore, the impact of dT-QX on the mitochondrial function was assessed using a fluorescence based mitochondrial superoxide imaging assay. Hep3B cells were investigated as the representative of cancer cell model because of the pronounced inhibition of DNA synthesis at early stage by dT-QX observed in anti-BrdU assay (Figure 4a).

**Figure 5:**
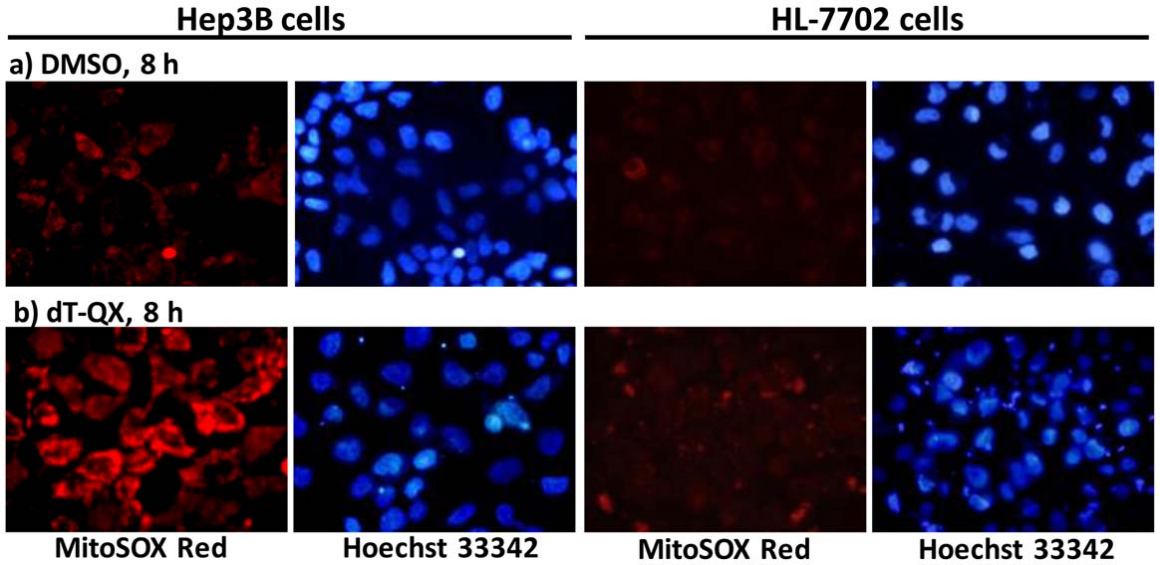
Fluorescence staining of mitochondrial superoxide production upon treatment. Cancer cell line Hep3B or normal HL-7702 cells were treated with DMSO or 50 µM dT-QX for 8 h. The level of mitochondrial superoxide was detected with MitoSOX mitochondrial superoxide indicator (red color). Cell nuclei were counter-stained with Hoechst dye (blue color).

Significant red fluorescence indicative of superoxide production was detected in Hep3B cells after treatment of 50 µM dT-QX for 8 h as compared to that DMSO (Figure 5). The cytosolic presence of red staining of superoxide was confirmed by counter-staining cell nuclei with a Hoechst dye. It was also found that treatment of Hep3B cells with dT-QX for a shorter time such as 3 or 5 h did not induce significant red fluorescence, suggesting the production of mitochondrial superoxide was accumulative and was likely due to the inhibition of cellular DNA synthesis. In addition, a positive control with a Mn(III)-chelator acting as a NADPH/GSH:O_2_^−^ oxidoreductase in cells [28] resulted in a similar level of red fluorescence in Hep3B cells to that treated with dT-QX (see Figure S8). In contrast, low background red fluorescence was observed in human normal HL-7702 cells treated with dT-QX, similar to that treated with DMSO (Figure 5). Thus, these results suggested that dT-QX could cause mitochondrial dysfunction by inducing mitochondrial superoxide stress in cancer cells following the inhibition of DNA synthesis.

**Figure 6:**
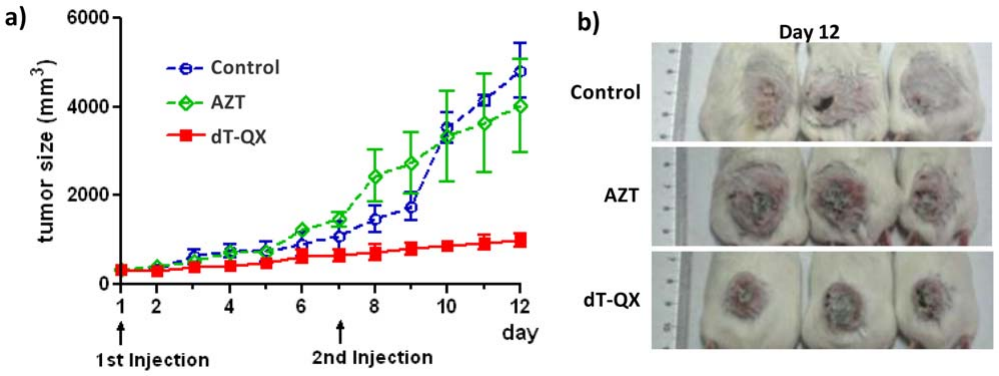
Growth inhibition of subcutaneous tumors by dT-QX in mice. Subcutaneous tumor was established by injecting H22 cell at the lower back of BALB/c mice subcutaneously (3×10^6^ cells per mouse). After tumors reached an average size of (8×8 mm, 340 mm^3^), mice (3 per group) were injected with 100 µL of saline (with 0.1% DMSO), AZT (50 µM in sterile PBS with 0.1% DMSO) or dT-QX (50 µM in sterile PBS with 0.1% DMSO) on the tumor site on day 1 and day 7. The tumor growth and body weight were monitored daily. On day 12 following the first injection, all mice were euthanized and images of tumors were recorded. a) Growth profile of average tumor size over the 12-day treatments of each treatment group; b) Images of tumors of each treatment group on day 12 after first injection.

A preliminary in vivo antitumor study with dT-QX was carried out in a mouse model. Subcutaneous tumors were established with murine H22 liver cancer cells [29]. Tumors were allowed to reach an average size of 8.8×8.8 mm. The relative low solubility of dT-QX in PBS solution limited the route of administration to be intra-tumor injections. Thus, AZT or dT-QX (100 µL×50 µM each) was injected directly into tumors on day 1 and day 7. Regardless of treatments, neither mice died nor was there a significant body weight loss observed during the 12-day study. The tumor growth profile over 12 days after the first injection is shown in Figure 6a. Tumors in mice treated with AZT grew rapidly, similar to those of negative control (saline); whereas the growth of tumors was significantly inhibited in dT-QX treated mice (Figure 6a). On day 12 after the first injection, the sizes of tumors in mice injected with dT-QX were clearly smaller than those with AZT or saline (Figure 6b). Statistic analysis also confirmed that the treatment of dT-QX was significantly different from the other two (*P*<0.01). These results suggested that intra-tumor injection of dT-QX at a low dosage (equivalent to 0.13 mg/kg per mouse) was quite effective at inhibiting the growth of subcutaneous tumors in a mouse model.

In conclusion, dT-QX as a novel thymidine analog exhibited an excellent selective cytotoxicity toward a variety of cancer cells but not on normal human liver HL-7702 cells. The selectivity was achieved through selective inhibition of cellular DNA synthesis by dT-QX, resulting in mitochondrial superoxide stress which may lead to mitochondrial dysfunction. The covalent linkage with 3′-deoxythymidine uniquely directed the cytotoxicity of phenylquinoxaline moiety more toward cancer cells than normal liver cells. Preliminary in vivo study also demonstrates that dT-QX could be a potential drug candidate for anti-cancer agents. dT-QX is the first molecule of its kind that exhibits this high selective cytotoxicity and excellent in vivo potency. Considering the highly amendable structural constituents for better anti-cancer activity and low hepatotoxicity, the potential of dT-QX as a lead compound for anti-cancer drug development is therefore very promising.

## Supporting Information

Figure S1 **NMR spectra of compound 2.**
(TIF)

Figure S2 **NMR spectra of compound 3.**
(TIF)

Figure S3 **NMR spectra of compound 4.**
(TIF)

Figure S4 **NMR spectra of compound dT-QX.**
(TIF)

Figure S5 **Electrospray mass analysis of dT-QX.**
(TIF)

Figure S6 **NMR spectra of compound 5.**
(TIF)

Figure S7 **NMR spectra of Pip-QX.**
(TIF)

Figure S8 **Levels of mitochondrial superoxide production in Hep3B cells upon MnTmPyP and/or dT-QX treatment for 8 h.** Fluorescent images of cells were captured after staining with MitoSOX Red mitochondrial superoxide indicator solution.
(TIF)
