# Supplementary figures and images for: Design, Synthesis, and In Vitro and In Vivo Biological Studies of a 3′-Deoxythymidine Conjugate that Potentially Kills Cancer Cells Selectively
Source: PLoS One. 2012 Dec 26;7(12):e52199. doi: 10.1371/journal.pone.0052199 (PMC3530607; doi:10.1371/journal.pone.0052199)

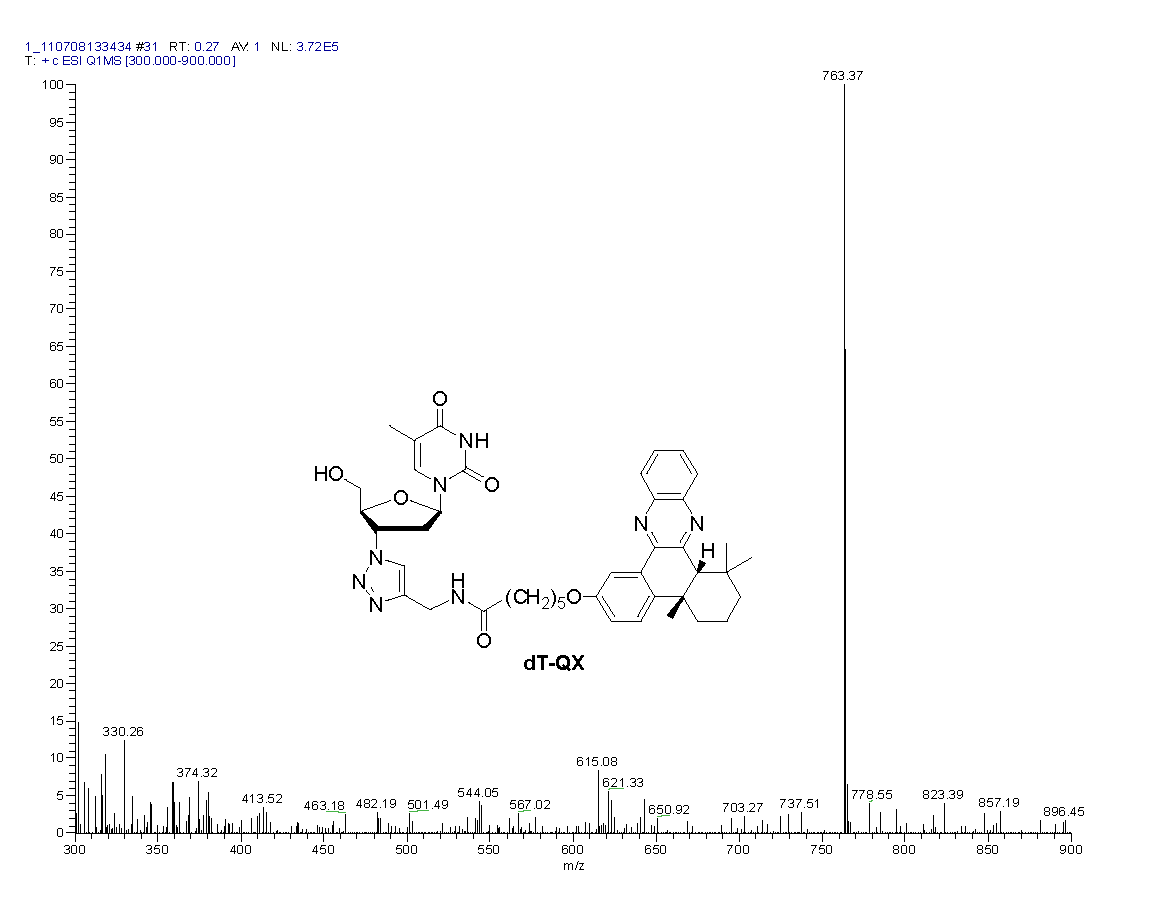

Supplement: Figure S5 — Electrospray mass analysis of dT-QX. (TIF) [file pone.0052199.s005.tif]

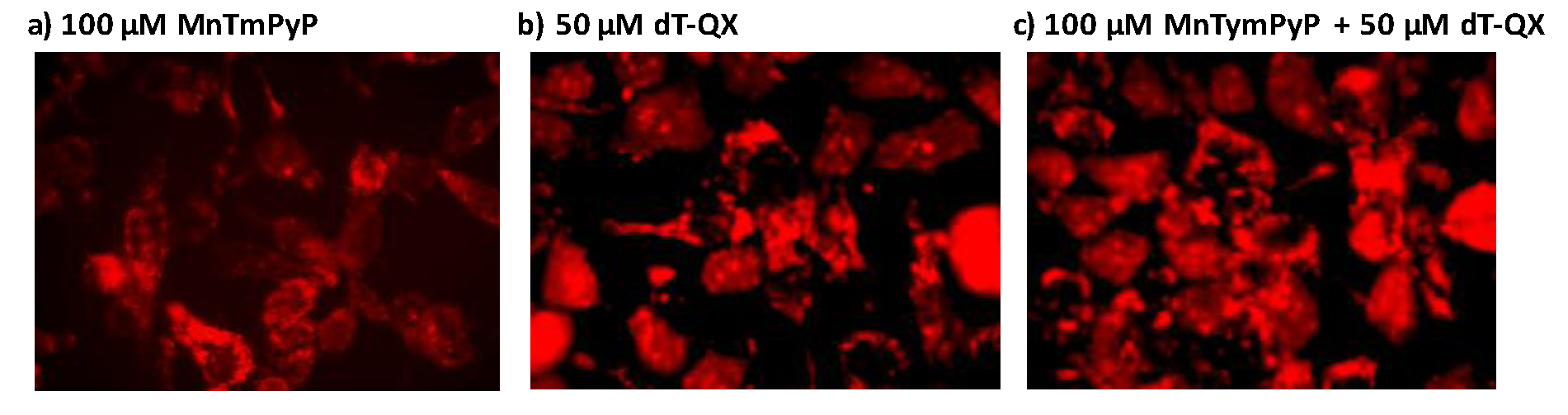

Supplement: Figure S8 — Levels of mitochondrial superoxide production in Hep3B cells upon MnTmPyP and/or dT-QX treatment for 8 h. Fluorescent images of cells were captured after staining with MitoSOX Red mitochondrial superoxide indicator solution. (TIF) [file pone.0052199.s008.tif]
